# Targeted therapy against EGFR and VEGFR using ZD6474 enhances the therapeutic potential of UV-B phototherapy in breast cancer cells

**DOI:** 10.1186/1476-4598-12-122

**Published:** 2013-10-20

**Authors:** Siddik Sarkar, Shashi Rajput, Amit Kumar Tripathi, Mahitosh Mandal

**Affiliations:** 1 School of Medical Science and Technology, Indian Institute of Technology Kharagpur, Kharagpur-721302, West Bengal, India

**Keywords:** Angiogenesis, Apoptosis, Breast cancer, Tyrosine kinase inhibitor (TKI), Epidermal growth factor (EGF) receptor (EGFR), UV-B phototherapy, Vascular endothelial growth factor (VEGF) receptor (VEGFR)

## Abstract

**Background:**

The hypoxic environment of tumor region stimulated the up regulation of growth factors responsible for angiogenesis and tumor proliferation. Thus, targeting the tumor vasculature along with the proliferation by dual tyrosine kinase inhibitor may be the efficient way of treating advanced breast cancers, which can be further enhanced by combining with radiotherapy. However, the effectiveness of radiotherapy may be severely compromised by toxicities and tumor resistance due to radiation-induced adaptive response contributing to recurrence and metastases of breast cancer. The rational of using ZD6474 is to evaluate the feasibility and efficacy of combined VEGFR2 and EGFR targeting with concurrent targeted and localized UV-B phototherapy *in vitro* breast cancer cells with the anticipation to cure skin lesions infiltrated with breast cancer cells.

**Materials and methods:**

Breast cancer cells were exposed to UV-B and ZD6474 and the cell viability, apoptosis, invasion and motility studies were conducted for the combinatorial effect. Graphs and statistical analyses were performed using Graph Pad Prism 5.0.

**Results:**

ZD6474 and UV-B decreased cell viability in breast cancers in combinatorial manner without affecting the normal human mammary epithelial cells. ZD6474 inhibited cyclin E expression and induced p53 expression when combined with UV-B. It activated stress induced mitochondrial pathway by inducing translocation of bax and cytochrome-c. The combination of ZD6474 with UV-B vs. either agent alone also more potently down-regulated the anti-apoptotic bcl-2 protein, up-regulated pro-apoptotic signaling events involving expression of bax, activation of caspase-3 and caspase-7 proteins, and induced poly (ADP-ribose) polymerase resulting in apoptosis. ZD6474 combined with UV-B inhibited invasion of breast cancer cells *in vitro* as compared to either single agent, indicating a potential involvement of pro-angiogenic growth factors in regulating the altered expression and reorganization of cytoskeletal proteins in combinatorial treated breast cancer cells. Involvement of combination therapy in reducing the expression of matrix metalloprotease was also observed.

**Conclusions:**

Collectively, our studies indicate that incorporating an anti-EGFR plus VEGFR strategy (ZD6474) with phototherapy (UV-B), an alternative approach to the ongoing conventional radiotherapy for the treatment of infiltrating metastatic breast cancer cells in the skin and for locally recurrence breast cancer than either approach alone.

## Background

Conventional radiotherapy (RT) using X-rays and γ-rays is used for the treatment of cancers and may be used as primary or in adjuvant settings. Treatment with radiation after breast cancer surgery as well as combined treatment of radiation and chemotherapy is anticipated to improve cancer treatment. Previous studies showed that adding radiation to breast cancer treatment doesn’t just lower a woman’s risk of having a relapse, it also improves survival [1]. However, radiation is related to potentially serious side effects including ischemic heart disease and pneumonitis, sterility [2-4]. Moreover radiotherapy led to development of radiation-induced adaptive response that contributes recurrence and metastases of breast cancer by upregulating Epidermal growth factor (EGF) receptor (EGFR) and vascular endothelial growth factor (VEGF) receptor (VEGFR) related proteins [5,6]. This led to the development of alternative form of RT, popularly known as phototherapy. It is based on the old concept of transfer of light energy or photons to form intermediates, which resulted in the consumption of oxygen. This reaction resulted in the formation of singlet oxygen or reactive oxygen species (ROS). These ROS are extremely toxic and have very short half-life, thus affecting the adjacent cells without affecting the surrounding tissues. Ultraviolet radiation mainly UV-B (290–320 nm) having ~ 4 eV energy will be sufficient to perform chemical reactions either forming DNA photo-adducts or ROS [7]. UV-B phototherapy is widely used for treating various skin disorders with minimal systemic toxicities and side effects [8]. Though UV-B has its limitation in reaching to the deeper tissues and organs, but the development of LASER technology along with fiber optic catheters guided by non-invasive imaging techniques e.g., Magnetic resonance imaging, ultrasound imaging makes feasible for the interstitial UV-B phototherapy to act in periphery as well deep tissues and organs harboring the tumor cells. In order to achieve the selective destruction of the target area, tumor specific photosensitizers are either applied locally or intravenously where light can be applied over the accumulated photosensitizers/UV-sensitizers using minimally invasive fiber optic catheters guided by imaging devices. DNA being the intrinsic UV-photosensitizers can form photo-adducts and pyrimidine dimers by the introduction of UV-B radiation, which generally halted the cell cycle progression in the S phase of the cell cycle and induced apoptosis. The dual selectivity of phototherapy due to preferential localization of photosensitizers [9] or UV sensitizers [10] only to malignant tissues, and restriction of photo-activation only in the limited zone of irradiation makes it an alternative therapy to pre-existing conventional RT. This phototherapy is considered as more targeted to destroy cancer cells or pathogens and less toxic to surrounding normal tissues than the conventional radiotherapy using ionizing radiation. To investigate the effects of UV-B phototherapy on breast cancer, we constructed a model in which cultivated breast cancer cells were exposed to different doses of UV-B radiation. UV-B radiation induces DNA photoproducts, such as pyrimidine dimers and (6–4) photoproducts [11,12]*.* Ionizing irradiation produces double- and single-strand DNA breaks. Cells respond to DNA photoproducts and DNA breaks by accumulation of functionally active p53 protein, a key event in response to cellular stress. The signaling pathways that trigger a cell to undergo apoptosis or alter the proliferation in response to UV radiation are not well understood. UV radiation activates p53, cell death receptor, ROS and induces mitochondrial release of cytochrome-*c*, leading to apoptosis [13,14]. Most of the clinical settings of UV-B used in treatment of skin disorders are principally based on the effect of UV-B on apoptotic effects of the irradiated cells.

RT alone, however, has not yielded ideal clinical outcome and it is often associated with increased production of EGF and VEGF that contributes to radio-resistance [15] by activating growth factor mediated pathways in squamous and mammary carcinoma cells [16-18]. Radiation exposure activates mitogen activated protein kinase (MAPK) pathway to a level similar to that observed by physiological growth stimulatory, EGF concentrations [16,17,19]. MAPK signaling has also been linked to increased expression of growth factors such as EGF, VEGF and transforming growth factor alpha (TGFα), leading to increased proliferative rate of surviving cells [20-22]. Growth factors such as VEGF and TGFα, in addition to a growth-promoting role *in vitro*, may also play an important role in the development of tumors *in vivo* due to their abilities in the promotion of angiogenesis. Like RT, UV radiation also activates VEGF signaling involving EGF/PI3K pathway, activates invasion by activating metalloproteinase [23-25]. Collectively, these findings argue that UV-B phototherapy may have a self-limiting effect on its toxicity via increased activity of EGFR and VEGFR and downstream signaling molecules such as the MAPK pathway. Thus, one interesting and promising research direction for improving the treatment of breast cancer could be a molecular-targeted therapy against EGFR and VEGFR in association with UV-B phototherapy.

Several studies demonstrate that the expression of EGF and EGFR is related with breast cancer growth, progression and aggressiveness and its overexpression is an indicative of poor prognosis [26,27]. VEGF is closely associated with the promotion of angiogenesis, increment of micro-vessel density and with early relapse in primary breast cancer [28], yet clinical trials of agents that target either EGF or VEGF signaling pathways alone have been disappointing. Some tumors may not respond well to EGFR inhibitors alone or may develop resistance to EGFR inhibitors. We hypothesized that targeting both the tumor and its vasculature by VEGF- and EGF-receptor (VEGFR, EGFR) blockade would improve breast cancer treatment and provide wider applicability particularly when combined with UV-B phototherapy. To test this hypothesis, we evaluated the feasibility of combining ZD6474, a dual tyrosine kinase inhibitor of VEGFR and EGFR [29-32], with UV-B radiation in breast cancer cell lines MCF-7, MDA-MB-231, MDA-MB-468 and T-47D. This preclinical work was undertaken to serve as a rationale to support the role of ZD6474 in the treatment of skin lesions infiltrated with metastatic breast cancer cells and also for the recurrence breast cancer with UV-B phototherapy, a promising treatment alternative to RT.

## Results

### Radiation (UV-B) suppresses cell viability of breast cancer cells

**Figure 1:**
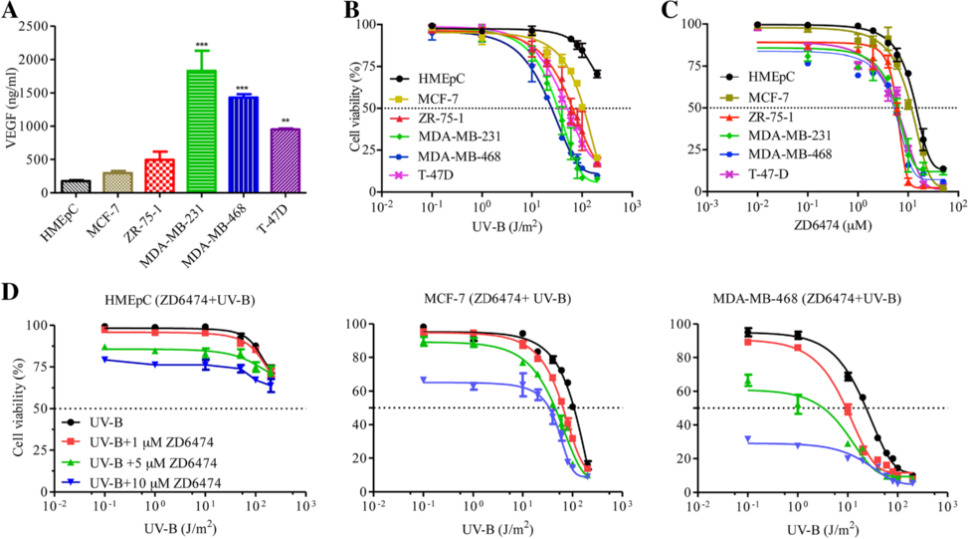
**Effect of radiation (UV-B) and ZD6474 is influenced by VEGF content in breast cancer cells. (A)** Cells were grown in serum-free CM for 48 h and VEGF was quantified by using ELISA. ** (p < 0.01), and *** (p < 0.001) indicates the increasing level of significance after performing un-paired t-tests between HMEpC and different breast cancer cells. Dose–response curve of **(B)** UV-B irradiated and **(C)** ZD-6474 treated breast cancer cells along with normal mammary HMEpC cells as analyzed by MTT assay after 48 h of post treatment. **(D)** Dose-dependent growth inhibition of breast cancer cells MCF-7, MDA-MB-468 and HMEpC by UV-B radiation in combination with ZD6474 as analyzed by MTT assay after 48 h of post treatment. Points, mean ± S. E. of three different experiments each performed in quadruplicate.

VEGF level was measured by using VEGF ELISA kit. The VEGF content of MCF-7, ZR-75-1, MDA-MB-231, MDA-MB-468 and T-47D was found to be 297.91 ± 32.62, 493.32 ± 33.31, 1829.11 ± 50.01, 1429.51 ± 40.01 and 948.21 ± 20.11 ng/ml respectively per 10^6^ cells (Figure 1A). The VEGF content of normal human mammary epithelial cells (HMEpC) was 110.00 ± 11.12 ng/ml, and is significantly lower than the breast cancer cells (MDA-MB-231 and MDA-MB-468). To compare the effect of UV-B on cell viability of breast cancer cells *in vitro*, MCF-7, ZR-75-1, MDA-MB-468, MDA-MB-231 and T-47D, and normal mammary epithelial HMEpC cells were treated with increasing doses of UV-B radiation (1–200 J/m^2^) and incubated in culture medium for 2 days. UV-B reduced cell viability in a dose-dependent manner and the cell growth inhibition was prominent mainly between UV-B doses of 10–100 J/m^2^ (Figure 1B). The IC_50_ values of UV-B irradiated MCF-7, ZR-75-1 MDA-MB-468, MDA-MB-231, and T-47D cells were 101.20 ± 3.86, 74.21 ± 4.01, 32.54 ± 2.67, 35.33 ± 1.23, and 42.12 ± 2.12 J/m^2^ respectively (Table 1), where as IC_50_ was found to be higher (>250 J/m^2^) as par as HMEpC was concerned. The VEGF level of MCF-7 is lowest among the cell lines but IC_50_ of UV-B in MCF-7 was found to be highest. MDA-MB-231 and MDA-MB-468 have highest level of VEGF (Figure 1A) and they were shown to be more radiosensitive to UV-B. Moreover the VEGF content of HMEpC is very less and hence showed reduced sensitivity towards UV-B mediated cell killing, indicating the role of UV-B phototherapy may be an alternative substitute for conventional radiotherapy. Based on the sensitivity to UV-B, we have chosen two cancer cell lines for further experiments i.e., MCF-7 (least sensitive) and MDA-MB-468 (most sensitive) to study the potentiating effect of UV-B influenced by ZD6474.

**Table 1:** ZD6474 potentiates UV-B action on breast cancer cells

**Cell line**	**Treatment regimen**	**IC**_**50**_**(J/m**^**2**^**)**	**PF**^**a**^
MCF-7	UV-B	101.20 ± 3.86	
	UV-B + ZD6474	59.20 ± 2.45	1.71
ZR-75-1	UV-B	74.21 ± 4.01	
	UV-B + ZD6474	41.26 ± 3.01	1.80
MDA-MB-468	UV-B	32.54 ± 2.67	
	UV-B + ZD6474	12.17 ± 2.49	2.67
MDA-MB-231	UV-B	35.33 ± 1.23	
	UV-B + ZD6474	15.12 ± 2.12	2.34
T-47D	UV-B	42.12 ± 2.12	
	UV-B + ZD6474	25.21 ± 2.12	1.67
HMEpC	UV-B	250.72 ± 9.12	
	UV-B + ZD6474	248.12	1.01

^a^Potentiation Factor (PF) indicates a quantitative measure of fold-sensitivity of UV-B radiation treated with 1 μM ZD6474 at a given effect compared with the sensitivity of UV-B alone.

### ZD6474 in combination with UV-B cooperatively inhibits growth *in vitro*

To evaluate potential cooperative interactions between dual tyrosine kinase inhibitor (TKI)-ZD6474 and UV-B (phototherapy), it was also necessary to study a dose response curve of ZD6474 in breast cancer cells. It was found that ZD6474 executed lesser toxicity in normal HMEpC as compared to breast cancer cells (Figure 1C). Thus it is anticipated that combinatorial effect of ZD6474 and UV-B will result in more efficient killing in breast cancer cells with minimal effect in normal breast epithelial cells. As a proof of principal, cells were treated with increasing doses of UV-B followed by treatment with 1 or 5 or 10 μM ZD6474. The effect of dual TKI-ZD6474 with UV-B showed combinatorial benefit. Treatment with ZD6474 in combination with UV-B resulted a leftward shift of the dose response curves (Figure 1D), indicating a greater cytotoxic effect. As the concentration of ZD6474 increases, there was further shift of dose response curves of UV-B radiation compared with combined effect of 1 μM ZD6474 and UV-B radiation. ZD6474 of 1 μM concentration potentiated the effect of UV-B radiation by more than 1.5-fold in all breast cancer cell lines (Table 1). There was > 75% cell viability when MCF-7 and MDA-MB-468 cells were treated with 5 μM ZD6474 alone. The decrease in cell number as well as the increase in cell death (apoptosis) was prominent at 100 J/m^2^ and 50 J/m^2^ in MCF-7 and MDA-MB-468 irradiated with UV-B alone. The radiation doses was further reduced to 50 and 25 J/m^2^ in MCF-7 and MDA-MB-468 respectively when 5 μM ZD6474 was added as combined treatment strategy to obtain the effect that was seen at higher radiation doses (Additional file 1: Figure S1). When breast cancer cells were treated with 10 μM ZD6474, the dose response curve showed lesser leftward shift indicating lesser synergistic or combinatorial effect which was expected as the dose of ZD6474 above the sublethal dose, a prime factor for any combinatorial therapy in cancer treatment. The most striking observation was there was no combinatorial effect observed in normal HMEpC (Figure 1D), further indicating the importance of combinatorial therapy in the cancer management.

### ZD6474 inhibits cell proliferation and induces apoptosis in combination with UV-B

**Figure 2:**
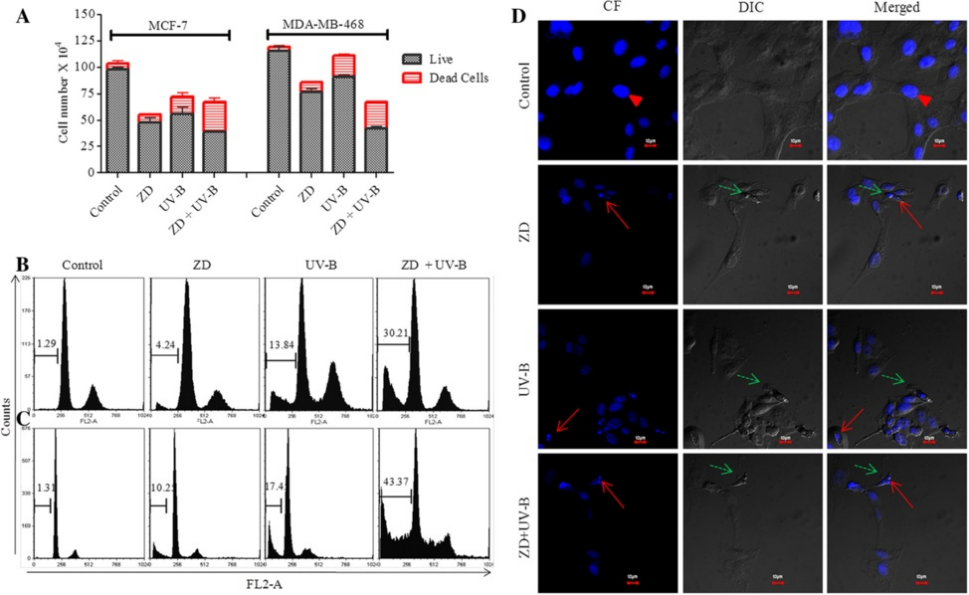
**ZD6474 enhances antiproliferative and apoptotic effects of UV-B radiation. (A)** The combinatorial effect of ZD6474 (ZD) and UV-B radiation in decreasing cell viability is attributed to both enhanced antiproliferative and apoptotic effects as shown by decreased cell counts by Trypan blue dye exclusion tests. **(B)** MCF-7 and **(C)** MDA-MB-468 cells were treated with ZD6474 (ZD) and/or UV-B radiation, and stained with PI for apoptosis measurements using flow-cytometry. The percentage of apoptotic cells is obtained by gating hypodiploid cells. **(D)** MDA-MB-468 cells were treated ZD and/or UV-B radiation and stained with DAPI, and observed under CLSM in confocal fluorescence (CF) mode and DIC mode and the merged (CF + DIC) image. Red arrows; apoptotic nuclei, green dashed arrows; membrane blebbing, Arrow heads; intact nuclei. Bars; 10 μM. Representative figures of three independent experiments.

Cell viability is a dynamic process that reflects a balance between cell proliferation and cell death. To define the contributory roles of proliferation and apoptosis in cell viability, Trypan blue dye exclusion tests and apoptosis based flow-cytometric assays were performed. Decreased cell viability was a consequence of both the growth inhibitory and apoptotic effects of ZD6474 when combined with UV-B (Figure 2A). There was >30% apoptosis in combinatorial-treated cells as compared to control cells, which was further confirmed by flow-cytometry. There were 30.2 ± 3.3, 43.3 ± 4.4% apoptosis in combination treatment as compared to 1.3 ± 0.5 and 1.4 ± 0.75% in untreated control of MCF-7 and MDA-MB-468 respectively. In contrast, there was less or no significant apoptosis observed when cells were treated with either agent alone (Figure 2A, 2B and 2C). Apoptosis was further confirmed by observing under CLSM. Formation of oligonucleosomes was easily recognized in MDA-MB-468 cell lines following combination treatment (Figure 2D). There was a prominent loss of cell membrane asymmetry, attachment, membrane blebbing and cytoplasm retraction, characteristic features of apoptosis, in combination treatment as compared to either agent alone or untreated cells.

### ZD6474 enhances the effect of UV-B in reducing mitochondrial membrane potential (ΔΨm)

**Figure 3:**
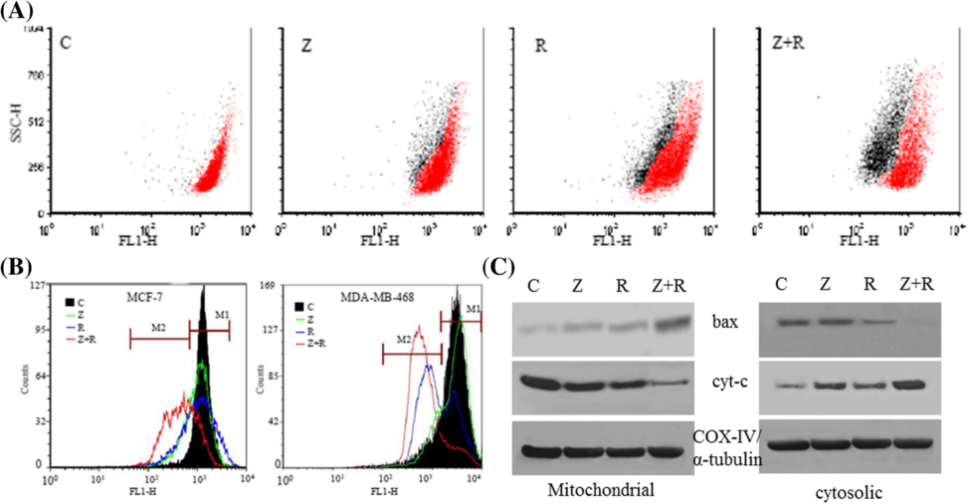
**Loss of mitochondrial potential (**Δ**Ψm) in ZD6474 and UV-B irradiated MDA-MB-468.** Cells were stained with Rh-123 after treatment with ZD6474 (Z) and/or UV-B radiation (R) along with untreated control **(C)**. Top panel represented **(A)** dot plot of treated MDA-MB-468. There was an evident change in ΔΨm in both MCF-7 and MDA-MB-468 in combination treatment as shown by the histogram plot **(B)**. Translocation of bax from cytosol to mitochondria vice versa for cytochrome*-c* translocation was observed in MDA-MB-468 in combination treatment as confirmed by western blotting **(C)**. COX-IV and α-tubulin was taken as loading control for mitochondrial and cytosolic fraction respectively.

To see the involvement of mitochondrial membrane potential (ΔΨm) in apoptosis induced by ZD6474 and/or UV-B radiation, fluorescence intensity and shift was monitored using potential-sensitive dye, rhodamine 123 (Rh-123) by flow-cytometry. In untreated control cells of MDA-MB-468, ΔΨm showed high potential (Figure 3A, and 3B). However, after 12 h of incubation with ZD6474 and/or UV-B, Rh-123 stained cells were separated into two populations (M1; higher membrane potential, M2; lower membrane potential) as shown in dot-plot and histogram-plot by fluorescent strength (Figure 3A, and 3B). There were 35.52 ± 5.87% and 45.93 ± 6.34% of MCF-7 and MDA-MB-468 cells showed a drastic reduction of the ΔΨm in combined treatment of ZD6474 and UV-B (Table 2). The reduction of ΔΨm was lower in ZD6474 treated cells as compared to the UV-B treated cells. In order to examine the involvement of bax and cytochrome-c translocation during this reduction in ΔΨm, mitochondrial fraction and cytosolic fraction of MDA-MB-468 cells treated with ZD6474 and/or UV-B for 24 h were collected and studied by western blotting. There was an evident of translocation of bax from cytosol to mitochondrial in UV-B irradiated MDA-MB-468 cells as the expression of bax is increased in mitochondrial fraction and subsequently decreased in cytosolic fraction as compared to untreated control cell (Figure 3C). There was no significant change of bax translocation in ZD6474 treated cells. But, the addition of ZD6474 in UV-B treatment strategy profoundly increased the expression of bax in mitochondrial fraction as compared to either agent alone. There was also change in expression of cytochrome-c in both subcellular fractions, indicating the involvement of reduced ΔΨm in association with cytochrome-c. Cytochrome-c was significantly decreased in mitochondrial fraction and increased in cytosolic fraction of cells treated with combined ZD6474 and UV-B as compared to either agent alone (Figure 3C), indicating its translocation from mitochondria to cytosol in combined treatment.

**Table 2:** **Mitochondrial membrane potential (**ΔΨ**m) of ZD6474 and/or UV-B treated breast cancer cells**

**Cell line**	**Treatment**	**M1 (%)**^**a**^	**M2 (%)**^**b**^
MCF-7	Control	95.37 ± 2.45	4.62 ± 2.45
	ZD6474	85.78 ± 3.16	14.22 ± 3.16
	UVB	81.07 ± 3.64	18.93 ± 3.65
	ZD6474 + UV-B	65.81 ± 3.89	35.52 ± 5.87
MDA-MB-468	Control	93.08 ± 1.36	6.92 ± 1.66
	ZD6474	87.47 ± 1.04	12.53 ± 1.27
	UVB	69.33 ± 2.92	30.66 ± 1.15
	ZD6474 + UV-B	54.06 ± 5.08	45.93 ± 6.34

^a^Percentage (%) cells in M1 population was calculated by gating cells at higher membrane potential. ^b^Percentage (%) cells in M2 population was calculated by gating cells at reduced membrane potential.

### ZD6474 enhances the downstream activation of Caspase-3 and Caspase-7 by UV-B radiation

**Figure 4:**
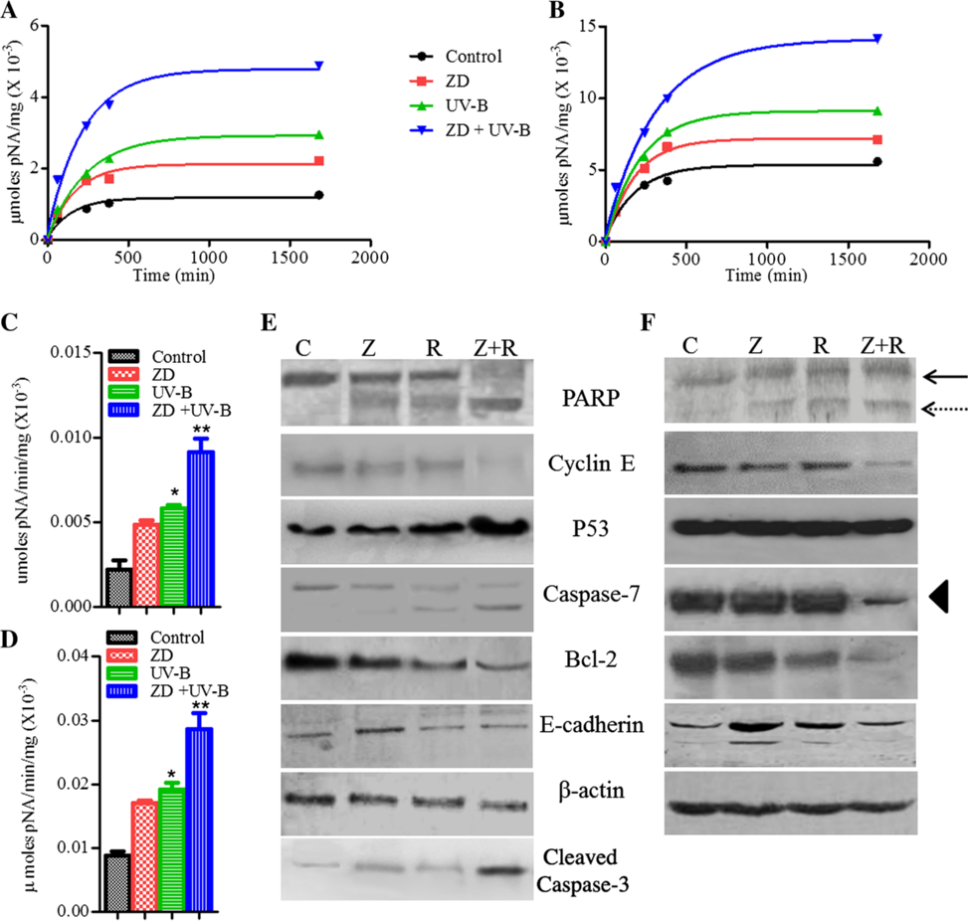
**ZD6474 modulates UV-B action by altering the expression of caspases and apoptotic proteins.** Time vs. pNA formation graphs of **(A)** MCF-7 and **(B)** MDA-MB-468 treated ZD6474 (ZD) and/or UV-B radiation for 48 h was studied by monitoring the spectrophotometric readings at 405 nm in order to study the Enzyme kinetics of caspase-3/7 using Ac-DEVD-pNA as substrate. Specific activity of Caspase-3/7 was studied for **(C)** MCF-7 and **(D)** MDA-MB-468. Bars (mean ± S.E, n = 3), and * (p < 0.05), ** (p < 0.01) represent levels of significance with respect to control. Western blotting of **(E)** MCF-7 and **(F)** MDA-MB-468 cells treated with ZD6474 (Z) and/or UV-B (R) for 48 h and probed with anti-PARP, cyclin E, caspase-3, casapse-7, bcl-2, E-cadherin. β-actin protein expression was used as an internal probe for equal loading. Representative of three independent experiments. Intact PARP, complete arrow; cleaved PARP, dashed arrow; block filled arrow, caspase-3 (MDA-MB-468).

To see the involvement of caspases downstream of mitochondrial pathway, casapse 3/7 activity assays of MCF-7 and MDA-MB-468 cells treated with ZD6474 and/or UV-B for 48 h were performed using acetyl-Asp-Glu-Val-Asp p-nitroanilide (Ac-DEVD-pNA) as the substrate. The rate of decomposition of Ac-DEVD-pNA into p-nitroaniline (pNA) reflects caspase-3/7 activation. The plateau of the peak reflects the active form of caspase-3/7. The plateau was significantly higher in combination treatment of ZD6474 and UV-B in both MCF-7 and MDA-MB-468 as compared to either agent alone or untreated control cells (Figure 4A and 4B). The specific activity was calculated at the linear region of enzyme kinetics (time vs. formation of pNA) graph of caspase-3/7. In control untreated cells, the activity was very less and there was a slight increase in activity in MCF-7 and MDA-MB-468 treated with ZD6474. The activity is significant when it irradiated UV-B alone, but it is very significant when ZD6474 was added in the treatment strategy of UV-B irradiated MCF-7 and MDA-MB-468 (Figure 4C and 4D). Thus, ZD6474 enhances the activity of UV-B radiation in the formation of active caspases downstream of mitochondrial pathway.

### ZD6474 alters cell regulatory proteins and apoptotic proteins when used in combination with UV-B

To elucidate the molecular mechanism or the proteins involved in enhanced activity of combination treatment of ZD6474 and UV-B radiation, we sought to study both cell regulatory and apoptotic proteins. There were marked decreases in Cyclin E expression in combination treatment compared to control as well as cells treated with either ZD6474 or UV-B radiation alone, whereas Cyclin E levels were unchanged in cells treated with either agent as compared to control. Though the change of p53 expression was distinguishable in UV-B irradiated breast cancer MCF-7 cells, but more significant changes in p53 levels in combination treated breast cancer cells was observed (Figure 4E). There was no change in expression of p53 in MDA-MB-468 (data not shown), but increased in expression of p21 was noted in combined ZD6474 + UV-B treated MDA-MB-468 cells (Figure 4F). Next we investigated the effect of single and combination treatment on the expression of apoptotic proteins. Cleavage of poly (ADP-ribose) Polymerase (PARP) was observed in MCF-7 and MDA-MB-468 cells treated with either of ZD6474 or UV-B as compared to control. The cleavage was more profound in combination treatment as there was increased expression of the 85-Kd fragment (cleaved PARP) with almost absence of the 116-Kd fragment (uncleaved PARP). There was a decrease in anti-apoptotic bcl-2 expression (Figure 4E and 4F). There was a noticeable decrease of pro-caspase-3 in MDA-MB-468 following combination treatment, indicating the formation of activated p11 and p17 caspase-3 in MDA-MB-468 cells (Figure 4F). Caspase-3 is absent in MCF-7, indicating a role of other effector caspases. There was decreased expression in pro-caspase-7 (35-Kd) and increased formation of active caspase-7 (20-Kd) in combination-treated MCF-7 cells (Figure 4E).

### ZD6474 inhibits cell migration when used in combination with UV-B radiation

**Figure 5:**
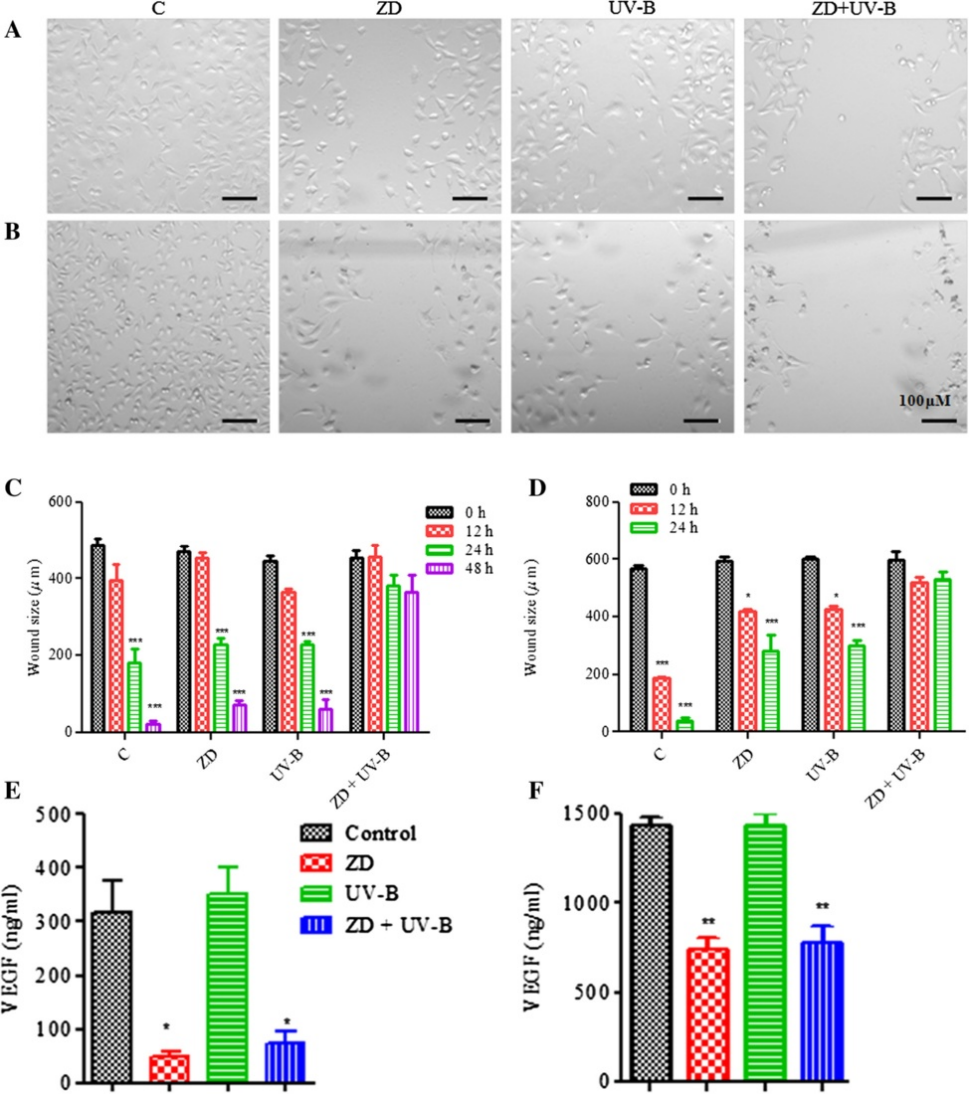
**ZD6474 inhibits migration in breast cancer cells in combination with UV-B by inhibiting VEGF signaling pathway.** Scratching across a cell monolayer on a 6-well culture plate created a wound and the width of the wound was recorded prior to treatment with ZD6474 and/or UV-B and after indicated h post-treatment. Photomicrograph of ZD6474 and/or UV-B treated **(A)** MCF-7 and **(B)** MDA-MB-468 cells after 48 h and 24 h post-treatment respectively. Scale bar, 100 μM. Bars, S.E., three random widths along the wound at indicated h post-treatment of **(C)** MCF-7 and **(D)** MDA-MJ B-468 cells. P-values were calculated by using un-paired t-test of the same treatment groups (prior and after stipulated h post-treatment). Breast cancer cells were treated with ZD6474 and/or UV-B and incubated in serum-free CM for 48 h. VEGF level of **(E)** MCF-7 and **(F)** MDA-MB-468 was determined by ELISA. All data were derived from a minimum of three independent experiments using different cell preparations. *P < 0.05, comparing levels of untreated control with treated cells.

Tumor cell migration is a critical factor in the formation of solid tumors and is necessary for their spread to distant organs. The process of metastasis requires changes in cell adhesion, increased cell migration, and angiogenesis. To determine the effect of ZD6474 and/or UV-B on migration, *in vitro* wound (scratch) assays were performed in both MCF-7 and MDA-MB-468 cultures. The size of the wound (scratch) before treatment was 487.60 ± 9.76 (mean ± S.E.), which was decreased to 180.37 ± 10.33, 228.00 ± 15.11, 227.00 ± 9.07 and 390.30 ± 25.36 for control, ZD6474, UV-B and combined ZD6474 and UV-B treatment in MCF-7 cells after 24 h post-treatment. In the case of MDA-MB-468, the size of the wound (scratch) prior to treatment was 568.70 ± 15.47, which was decreased to 39.69 ± 10.69, 279.30 ± 25.12, 300.70 ± 18.32 and 529.80 ± 28.90 for control, ZD6474, UV-B and combined ZD6474 and UV-B treatment, respectively, 24 h post-treatment. These results showed that ZD6474 in combination with UV-B effectively blocked cell migration of MCF-7 and MDA-MB-468 cells (Figure 5A and 5B) and inhibited wound healing, as there was no significant change in wound size of both MCF-7 and MDA-MB-468 cells 48 h and 24 h post-treatment respectively with the combination of ZD6474 and UV-B as compared to the initial time of treatment. The cell migration was more prominent in MDA-MB-468 as compared to MCF-7 as the scratch was almost completely filled after 24 h in MDA-MB-468 as compared to 48 h post-treatment in MCF-7. There was also significant change in wound size in MDA-MB-468 cells after 12 h as compared to 24 h post-treatment in MCF-7 (Figure 5C and 5D). Accordingly, the EGFR and VEGFR-2-TKI ZD6474 may be an effective tool in inhibiting tumor formation as well as blocking breast cancer invasion and potentially metastasis. Additionally, there was an increase in E-cadherin expression in MCF-7 and MDA-MB-468 cells after treatment with either ZD6474 or UV-B (Figure 4E and 4F), suggesting a role in cytoskeletal reorganization and stabilization, but the decrease in expression of E-cadherin in combination treatment may be a consequence of induction of apoptosis. Next we investigated the role of ZD6474 and/or UV-B radiation in the production of VEGF, proangiogenic factor, responsible for migration and invasion of breast cancer cells. VEGF secretion in the serum-free culture conditioned medium was measured using ELISA after 48 h post-treatment of breast cancer cells with ZD6474 and/ or UV-B radiation. It was found that ZD6474 inhibits VEGF secretion by ~6-fold as compared to untreated MCF-7 (Figure 5E). Though there was upregulation of VEGF secretion in MCF-7 irradiated UV-B, but the change was not significant (p = 0.713, n = 3). It was found that ZD6474 inhibited VEGF secretion significantly in UV-B irradiated MCF-7 (p = 0.003) as compared untreated MCF-7. There is also decrease in secretion of VEGF in ZD6474 treated MDA-MB-468 as compared to untreated cells (p = 0.001), and the decrease is also significant (p = 0.003) in combined ZD6474 + UV-B treated MDA-MB-468 cells (Figure 5F).

### ZD6474 in combination with UV-B induces cytoskeleton reorganization in breast cancer cells

**Figure 6:**
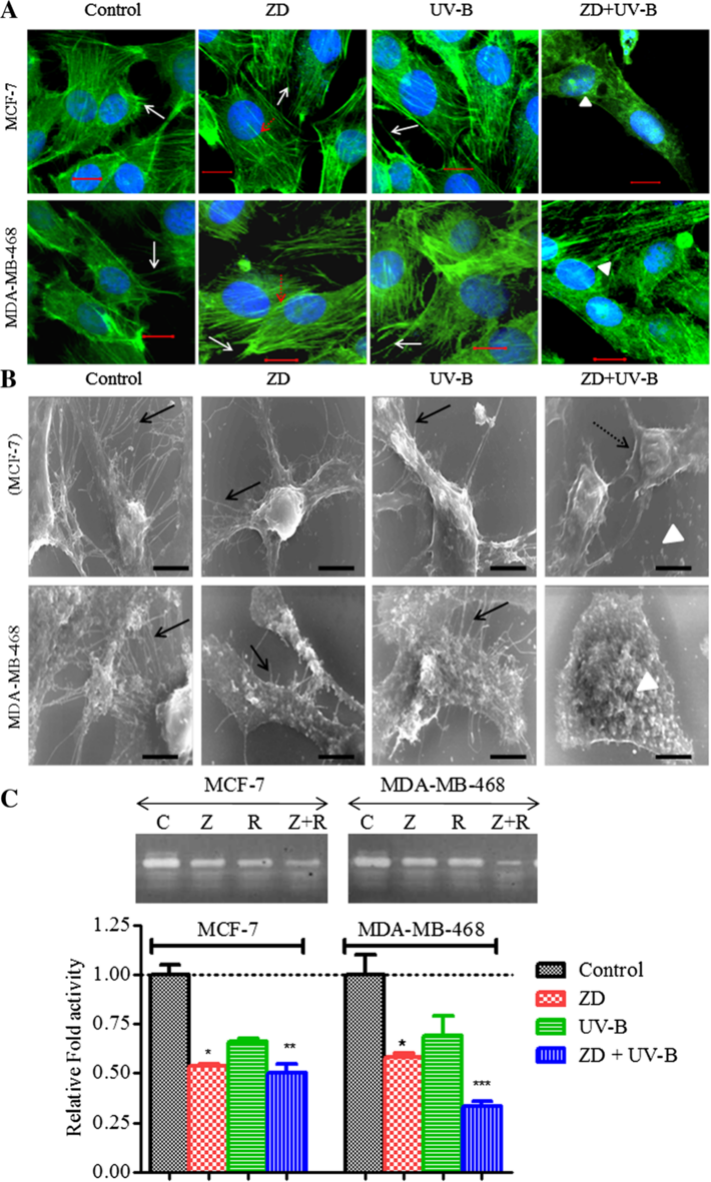
**ZD6474 in combination with UV-B alters cytoskeleton organization and extracellular MMP-9.** MCF-7 and MDA-MB-468 cells were treated with ZD6474 (ZD) and/or UV-B for 24 h, and then were immunofluorescently labeled using fluorescently-labeled phalloidin (F-actin-binding protein, green) and DAPI (DNA binding dye, blue). ZD and UV-B effectively blocked cell spreading lamellipodia (white arrow) and induced thick and stress actin fiber (red dashed arrow), whereas combination treatment lead to contraction of cytoplasm and F-actin rings around the periphery of the nucleus as observed under **(A)** CLSM (white filled arrow head). Bars, 10 μM. ZD blocked lamellipodia and filopodia formations (black arrow), which when combined with UV-B lead to contraction of cytoplasm (black dashed arrow) and formation of membrane blebs and apoptotic bodies (white filled arrow head) in addition with drastic loss of lamellipodia-like structures as evident from the photomicrograph of the surface of breast cancer cell studied under under **(B)** scanning electron microscope (SEM**)**. Bars, 10 μM. ZD6474 (ZD/Z) in combination with UV-B (R) effectively inhibits MMP-9 activity as evident from **(C)** Zymograph of CM of MCF-7 and MDA-MB-468 cells treated with ZD and/or UV-B.

To understand and correlate the effects of ZD6474 and/or UV-B in cell migration and motile phenotypes, we used confocal laser scanning microscopy (CLSM) to study cytoskeletal remodeling and generation of membrane protrusions, such as pseudopodium, filipodia and ruffle formation. ZD6474 lead to reorganization of F-actin structure. Long stressed F-actin filaments were observed across the cell in ZD6474 as compared to control cells (Figure 6A). Stress fibers were not prominently visible in UV-B treated cells as compared to ZD6474. In contrast, the combination of ZD6474 and UV-B produced F-actin rings exclusively in the perinuclear zone and the contraction of cytoplasm, indicating apoptosis was noticeable. ZD6474 and UV-B blocked membrane protrusions, such as microspikes, filopodia and lamellipodia formation, which was almost absent in MCF-7 and MDA-MB-468 following combination treatment with ZD6474 and UV-B (Figure 6A). The loss and dramatic collapse of cytoskelatal structure following combination treatment may be a consequence of induction of apoptosis. In the study of cancer therapy and invasion, high resolution SEM is a vital tool for analysis of expression of microspikes like lamellipodia and fillipodia, a cytoskeleton protein involved in the movement of cancer cells. The ultra-structure of cells was observed by FE-SEM. The images of untreated control MCF-7 and MDA-MB-468 showed the appearance of lamellipodia and fillipodia (Figure 6B) in consistent with previous results observed under CLSM. Interestingly, membrane blebs, and apoptotic bodies were observed in combined ZD6474 and UV-B, indicating apoptosis. Microspike-like protrusions were reduced drastically in MCF-7 and MDA-MB-468 cells treated with ZD6474, and it was completely lost in combination treatment, reflecting the enhanced activity of ZD6474 in reducing cell migration of breast cancer cells irradiated with UV-B (Figure 6B). Next, we investigated the effect of ZD6474 and UV-B on the secretion of MMP-9, which is believed to play an important role in tumor invasion. Zymographic analyses showed ZD6474 inhibits Matrix metalloprotease (MMP-9) activity (Figure 6C). Apart from its anti-EGF and VEGF effect in inhibiting tumor cells, it can also inhibit metastasis and spread of breast cancer cells by inhibiting MMP. Though decrease in MMP-9 activity was observed in case of UV-B irradiated cells, but it was not significant. The addition of ZD6474 enhanced its anti-metastatic potential by > 2-fold with respect to untreated control (Figure 6C).

## Discussion

Locally advanced breast cancer constitutes 30-60% of breast cancer cases and remains a clinical challenge as the majority of patients with this diagnosis develop distant metastases despite appropriate and preexisting radiotherapy and surgery [33]. Locally advanced breast cancers are often associated with higher expression of growth factors EGF, VEGF that are associated with shorter relapse free survival or over-all survival and aggressiveness of the disease [34,35]. Thus, there is a requirement of developing non-toxic, more effective novel therapeutic approach to combat this loco-regional recurrence of breast cancer, particularly for the patients treated prior with RT. These studies were initiated to further understand the role of VEGF with aggressive nature of breast cancer cells *in vitro*. MDA-MB-231 and MDA-MB-468 showed higher expression of VEGF and are more aggressive as compared to T-47D and MCF-7, least aggressive of the four cell lines. IC_50_ was > 40 J/m^2^ in both MDA-MB-468 and MDA-MB-231 cells. IC_50_ was < 40 J/m^2^ in T-47D and the IC_50_ ~100 J/m^2^ for MCF-7 irradiated cells (Figure 1). It indicates that the higher levels of VEGF in breast cancer cells *in vitro* are more sensitive to phototherapy (UV-B), and the lesser expression of VEGF will help in the normal mammary endothelial cells to escape the UV-B phototherapy, an important factor to consider for the safety of UV-B phototherapy in breast cancer treatment. Previous findings have shown that higher levels of EGF, VEGF and their cognate receptors were found to be the predictor of radio-response as compared to non-responders [34]. We observed similar findings with UV-B phototherapy. Previously it was also noticed that UV induced DNA damage resulting in cell death is dependent on nuclear excision repair protein (NER) protein [36]. In order to check the effect of UV-B radiation on nucleotide excision repair (NER) pathway, we have checked the level of XPA and ERCC1 expression (Additional file 2: Figure S2), and found that the sensitivity of UV-B in mediating cell death doesn’t completely depend on the level of NER pathway involved proteins i.e. XPA and ERCC1. Thus, the additional pathway might be involved in UV-B mediated cell death. It was shown that apart from DNA damage induced cell death by UV-B, death receptor pathway, decrease in mitochondrial potential (ΔΨm) and ROS are also involved in cell death [14,37,38]. Moreover, it was earlier reported that the “window “ of operating NER pathway is confined to low doses of UV-B where as at high doses of UV-B, NER involvement is not observed, and the apoptotic mechanism dominates over NER pathway [39]. To date, the pathways involving UV-B mediated apoptosis is not well elucidated and interestingly we have found a strong correlation of UV-B sensitivity and VEGF expression in breast cancer cells. Considering also the fact that UV-B lead to VEGF overexpression resulting in radio-resistance, it prompted us to investigate the role of anti-VEGF agent in sensitizing UV-B phototherapy mediated apoptosis in breast cancer cells.

RT is effective modality of treatment widely used for treating higher staging or locally advanced breast cancers [40]. Although widely used, a need remained to improve the cure rate by RT alone. The treatment based on chemotherapeutic agents paclitaxel, doxorubicin to RT in non-operable and recurrent disease, was found to be of good efficacy [41-43]. The cytotoxicity of chemotherapeutic agents, however, is not limited to tumor cells because treatment of tumors with these agents can result in significant normal tissue toxicity. Thus, the current therapeutic challenge is to optimize available non-operative strategies by incorporating new non-cytotoxic agents into current therapeutic regimens of RT. These led to the development of antiangiogenic therapies or molecular targeted therapies (Tyrosine kinase inhibitors) that target specific receptors VEGFR in endothelium cells that forms capillaries and supplies nutrients for hundreds of tumor cells. Hence, targeting of the tumor vasculature should lead to a potentiation of the antitumorigenic effect [44]. Some recent preclinical studies suggest that the combination of RT and angiogenic blockade enhances the therapeutic potential of ionizing radiation by targeting both tumor cells and tumor vessels [45,46]. However, loco-regional recurrence of breast cancer after surgery and post-operative RT occurs around 10-20% and 5-8% respectively [25]. Thus, phototherapy utilizing the energy of photons in combination with photosensitizers can be used to direct the energy to generate ROS or DNA damage in the tissue specific manner seems to be a promising alternative for treatment of advanced breast cancer patients for whom the RT is limited due to prior therapies. There is a recent development of targeted phototherapy, photosensitizers [47,48] that further minimizes the toxicities associated with UV phototherapy. Ionizing radiation enhances both epithelial growth factor receptor (EGFR) and vascular endothelial growth factor (VEGF) expression, and similar results were obtained with UV radiation [23,49], which are a part of key pathways for tumor progression and radioresistance [16]. It was also noticed that there was positive correlation between VEGF expression and ZD6474 sensitivity in decreasing cell proliferation as shown in Figure 1C. Thus, it supports the rational of combining UV-B radiation and ZD6474 in treating breast cancer cells. Moreover, it was found that 5-flurouracil, an anti-cancer drug with ionizing radio-sensitization activity, also enhanced the UV-B mediated apoptosis in breast cancer [50]. Previously it was shown that dual targeting of EGFR and VEGFR in combination with RT enhanced antitumor activity of lung cancer *in vivo* as compared to either agent alone [51]. Considering these previous findings, it is likely that EGFR and VEGFR-TKI ZD6474, when combined with UV-B phototherapy, will improve tumor control and provide wider applicability. The mechanisms by which tumor response to UV-B radiation is enhanced by ZD6474, however, are not currently understood.

In our study using *in vitro* breast cancer cells MCF-7 and MDA-MB-468 that closely recapitulates breast cancer with lower and higher VEGF expression respectively, we found that ZD6474 substantially improved radio-response to UV-B in both cell lines. The radio-sensitivity to UV-B was > 2-fold in higher expressed VEGF producing MDA-MB-231 and MDA-MB-468 (Table 1) when treated with 1 μM ZD6474 in combination with UV-B. The mechanism underlying the decrease in cell viability following combination treatment with ZD6474 and UV-B was studied. The photomicrograph of MCF-7 and MDA-MB-468 irradiated with increasing doses of UV-B clearly demonstrated the involvement of apoptosis in decreasing cell viability (Figure 2) with lesser involvement of antiproliferative effects, which was further confirmed from cell counts using trypan blue dye exclusion assays. It was shown earlier that UV radiation induced apoptosis as compared to ionizing radiation that mainly induced cell cycle arrest in osteosarcoma *in vitro*[52]. Moreover the extent of DNA damage, cell type, and genetic alterations determined the cells/tissues response to radiation to undergo either apoptosis or cell cycle arrest. Hence, the elucidation of the mechanism of UV-induced apoptosis in breast cancer will be important to make a rational decision for combining UV-B radiation with chemotherapeutic agents or small inhibitors e.g., TKI. In contrast to UV-B, ZD6474 is more an antiproliferative agent than a cytotoxic agent at its lower concentration (<IC_50_). The enhanced activity of ZD6474 in decreasing cell viability may be contributed both due to antiproliferative and apoptotic effects of combination treatment (Figure 2). ZD6474 significantly potentiates the apoptotic activity of UV-B as shown by flow-cytometry (Figure 2). Formation of oligonucleosomes or fragmented DNA, membrane blebbing further confirmed that cell death was due to activation of the apoptotic pathway as shown in Figure 4. Our findings have shown that ZD6474 may improve the therapeutic index for UV-B phototherapy by enhancing tumor-specific cytotoxicity.

Non-cytokine-mediated cellular stress, such as UV or chemical treatment, can initiate apoptosis through mitochondrial release of cytochrome-*c*[13]. There was a significant change in mitochondrial membrane potential (ΔΨm) that is associated with release of cytochrome-*c* in cytosol, initiating the apoptotic pathway mediated by mitochondria. There was also change in bax translocation (Figure 3), further implying the involvement of mitochondria in stress signaling pathway induced by UV-B radiation [53]. It was also found that ZD6474 increased the active form of caspase-7 (effector caspase in MCF-7 as it is deficient in caspase-3) in UV-B irradiated cells. It was confirmed both by catalytic activity of caspase-7 and protein expression observed by western blotting. But the enhanced catalytic activity of ZD6474 induced UV-B irradiated MDA-MB-468 was found to be associated with increased expression of active form of casapse-3 (Figure 4). There was also a slight change in caspase-7 activity (data not shown) in ZD6474 induced UV-B irradiated MDA-MB-468 cells. These eventually led to the formation of apoptosome, a multi-protein complex containing cytochrome-*c*, Apaf-1, and pro-caspase-9 and finally activation of effector caspase-3/7 leading to apoptosis [54].

The molecular mechanism involving the enhanced activity of combination treatment was further investigated by western blotting. There was a decrease in cyclin E (Figure 4) expression following combination treatment as compared to untreated control and exposure to single agents alone, indicating cell cycle arrest at G_1_-S or synthetic phase in UV-B irradiated cells. UV-B radiation in presence of ZD6474 induced DNA damage irreparable that ultimately arrested the irradiated cells at synthetic S or G_1_-S phase of cell cycle [55]. There was a decrease in expression of cyclin E in ZD6474 induced UV-B irradiated cells which is in agreement with our prior findings [56]. The alteration of both cyclin D1 and cyclin E was associated with breast cancer progression, early relapse, poor prognosis and chemo-resistance to various cytotoxic agents [57-59]. There was an increase in expression of p53, and a decrease in anti-apoptotic bcl-2 protein in breast cancer cells treated with combined ZD6474 and UV-B (Figure 4). The increase in p53 expression after cytotoxic insults was obvious, which is in agreement with previous and recent findings [52,60]. Previous findings had shown that increase in p53 expression was mainly due to p53 stabilization in irradiated cells as compared non-irradiated cells or cells capable of DNA repair. It was also shown that there was more increased expression of p53 in UV-B irradiated cells as compared to X-ray irradiated cells, eventually leading to more apoptosis in the former irradiated cells. Though p53 level was unchanged in ZD6474 treated cells, but its addition in the treatment strategy of UV-B irradiated cells increased the cytotoxicity nature of the cells that lead to further insults in DNA damages as evident in cell viability and flow-cytometric assays which were in consistent with higher expression of p53 in combination treatment in wild type p53 MCF-7 cell line (Figure 4), and no such change was associated with mutant p53 bearing MDA-MB-468. Previous findings had shown that UV induced apoptosis via direct p53-E2F1-Bcl-2 pathway by downregulating Bcl-2 where as it can also induced apoptosis in p53 independent manner via direct effect of Bcl-2 regulation by pyrimidine dimers [61]. Thus, Bcl-2 might play an important role in UV-B induced apoptosis. So, we checked the Bcl-2 expression in combined therapy, and noticed that Bcl-2 was downregulated by UV-B radiation in cell lines expressing wild type p53 (MCF-7) and its mutant form (MDA-MB-468), indicating that UV-B induced apoptosis acts through both p53 dependent and independent pathways which is in agreement with prior findings [61,62].

Cell migration and invasion are crucial steps in the physiopathology of development of cancer and metastasis [63,64]. ZD6474 inhibited motility of breast cancer cells (Figure 5) that was further decreased when ZD6474 is combined with UV-B. It was found that 48 h was required to fill the scratch in MCF-7 as compared to 24 h in MDA-MB-468, which is in agreement with previous findings that MDA-MB-468 is more aggressive of the two due to higher content of VEGF (chemotactic growth factors) in the former. We found that ZD6474 decreased VEGF expression probably by downregulating PI3K pathway [65] that contributes to downregulation of VEGF transcription (Figure 5E, and 5F). Though not significant, but an increased in VEGF level was observed in both cell lines when treated with UV-B radiation. It might be due to the fact that the cytotoxic effects induced by UV-B dose that was used in the experiment inhibited VEGF expression probably. There are reports, which suggest that UV radiation is an inducer of VEGF [23,66]. Thus the addition of ZD6474 to UV-B radiation might be beneficial in inhibiting its proangiogenic related activities.

The decreased motility observed in these cells may have an effect on cytoskeletal and cell adhesion molecules induced by ZD6474. Motility depends on an ordered series of events that require cell polarization, membrane extension into a lamellipodium, filipodium, attachment of the leading edge to the substratum, traction by stress fibers formed from the leading edge, and release of the lagging end of the cell. ZD6474 decreased cellular lamellipodia and filopodia extrusions and resulted in an almost complete loss of these projections in combination treatment (Figure 6A, and 6B). ZD6474 also increased E-cadherin expression in both cell lines (Figure 4E). Thus, ZD6474 stabilized cytoskeletal structure and inhibited invasion and migration of cancer cells. This is consistent with earlier studies demonstrating that intermediate levels of adherence are needed for optimal migration and that increasing or decreasing adherence actually decreases the rate of cell migration [67,68]. Loss of actin organization is characteristic of many tumor cells. Our results suggest that ZD6474 stabilized stress actin filaments, characteristics of normal differentiated cells. In case of UV-B irradiated cells, the change was not significant but the combined treatment with ZD6474 and UV-B led to disorganized actin filaments due to increased apoptosis [69].

## Conclusions

Collectively, our studies support a therapeutic approach for loco-regional occurrence of breast cancer that includes treatment with a dual EGFR- and VEGFR-targeted agent plus UV-B phototherapy, particularly those for whom the use of RT is limited by prior therapies. In addition to inhibiting endothelial cell proliferation and angiogenesis by blocking VEGF-induced signaling, ZD6474 also inhibited cancer cell growth and induced apoptosis. ZD6474 enhanced UV-B action in inhibiting cell viability by inducing apoptosis of breast cancer cells *in vitro.* ZD6474 modulated the angiogenic properties of UV-B radiation. It also has the potential to inhibit cell migration and metastases. Considering the fact that UV-B phototherapy is already being practiced in clinics for skin lesions, and the preclinical success of dual TKI in combination therapy with various anti-cancer agents, these observations have considerable potential clinical relevance for patients with locally advanced breast cancer or skin lesions infiltrated by malignant breast tumor.

## Materials and methods

### Cell lines

Human breast cancer cell lines MCF-7, MDA-MB-231 and MDA-MB-468 were cultured in Dulbecco’s Modified Eagle’s Medium: Nutrient Mixture F-12 (Ham) (D-MEM/F-12) with 15 mM HEPES buffer, L-glutamine, pyridoxine hydrochloride, supplemented with 1.2 g Sodium bicarbonate (Invitrogen Corporation, CA), antibiotics (10,000 U/L penicillin and 10 mg/L streptomycin) (Himedia, Mumbai, Maharashtra India) and 10% fetal bovine serum (FBS) (Invitrogen, Grand Island, NY, USA). T-47D and ZR-751 cells were grown in RPMI-1640, supplemented with 10% FBS. Human Mammary Epithelial Cells (HMEpC) (Cell Applications, Inc., San Diego, CA, USA) and were grown as per as manufacturer instructions. Cells were incubated at 37°C in a 5% CO_2_ and 95% humidified incubator.

### Reagents

Stock solutions of 20 mM ZD6474 (AstraZeneca Pharmaceuticals, Macclesfield, United Kingdom) were dissolved in DMSO (Sigma-Aldrich, St. Louis, MO, USA), stored at −20°C, and diluted in fresh medium just before use. For Western blot analysis, the following antibodies were used: rabbit monoclonal anti-PARP, anti-E-cadherin, mouse monoclonal anti-cyclin E, anti-caspase-3, (Cell Signaling Technology, Beverly, MA, USA), mouse monoclonal anti-caspase-7 (BD Pharmingen, Franklin Lakes, NJ, USA), mouse monoclonal anti-β-actin (Sigma-Aldrich), mouse polyclonal anti-bcl-2, anti-bax, anti-p53, horseradish peroxidase-conjugated goat anti-rabbit IgG and goat anti-mouse IgG, alkaline phosphatase-conjugated goat anti-rabbit IgG and goat anti-mouse IgG (Santa Cruz Biotechnology, Santa Cruz, CA, USA). Chemiluminescent peroxidase substrate, BCIP/NBT, Propidium iodide (PI), 4′,6-diamidino-2-phenylindole (DAPI) and 3-(4,5-dimethylthiazol-2-yl)-2,5-diphenyltetrazolium bromide (MTT), acetyl-Asp-Glu-Val-Asp p-nitroanilide (Ac-DEVD-pNA), Gelatin A and Gelatin B (Sigma-Aldrich), and Fluorescein phalloidin (Molecular Probes™, Invitrogen Corporation, Eugene, Oregon) , were purchased from the indicated company. Stock solutions of PI and DAPI were prepared by dissolving 1 mg of each compound in 1 ml PBS and MTT in incomplete medium. The solution was protected from light, stored at 4°C, and used within 1 month. Stock concentrations of 10 mg/ml RNase A dissolved in water and 20 mM Ac-DEVD-pNA (Sigma-Aldrich) dissolved in DMSO were prepared and kept at −20°C.

### UV-B irradiation

For UV-B irradiation, the medium was removed from cells grown in cell culture plates or in 96-well tissue before UV exposure. Cells were exposed to UV-B using a UV cross-linker (Agilent Technologies, Inc., Stratagene, Santa Clara, CA, USA) equipped with 598 W tubes which emit most of their energy within the UV-B range (290–320 nm) with an emission peak at 312 nm [70]. Control cells were treated similarly by the same protocol, except for radiation. After irradiation, cells were re-incubated in culture medium with or without ZD6474.

### Evaluation of cytotoxicity of ZD6474 and/or UV-B irradiation

Cells were harvested in the logarithmic phase of growth; cell suspensions were dispensed (200 μl) into 96-well tissue culture plates at an optimized concentration of 1 × 10^4^ cells/well in complete medium. 24 h after seeding, cells were irradiated with UV-B (1–200 J/m^2^) after the removal of the medium, and then reincubated for 48 h in the medium with different concentration of ZD6474 along with control treatment (0.1% DMSO. Cell viability was measured by MTT dye reduction assay at 540 nm. The dose-effect curves were analyzed using Prism software (GraphPad Prism 5.0, San Diego, CA, USA). For all subsequent experiments 1 μM ZD6474 and 25 J/m^2^ UV-B dose was selected, until otherwise mentioned.

### Apoptosis measurement by flow-cytometry

To study the effect of combination treatment of ZD6474 and UV-B cells were irradiated with 25 J/m^2^ UV-B, followed by treatment with 1 μM ZD6474 for 48 h after seeding in 60-mm tissue culture plates. After treatment, both attached and floating cells were collected and washed in phosphate-buffered saline (PBS) and incubated in 70% ethanol, kept at −20°C overnight for fixation. Cells were centrifuged, washed and then incubated with PI solution (40 μg/ml PI, 100 μg/ml RNase A in PBS) at 37°C for 1 h. Apoptotic cells were determined by their hypochromic sub-diploid staining profiles. The distribution of cells in the different cell-cycle phases was analyzed from the DNA histogram using Becton-Dickinson FACSCalibur flow-cytometer and CellQuest software.

### Measurement of mitochondrial membrane potential (ΔΨm)

To measure mitochondrial transmembrane potential (ΔΨm), rhodamine 123 (Rh-123) were used [71]. MCF-7 and MDA-MB-468 cells were treated with ZD6474 and/or UV-B radiation for 12 h. After that cell were washed with PBS, and were stained with Rh-123 at the final concentration of 5 μg/ml for 30 min at 37°C. Samples stained with Rh-123 were subjected to flow-cytometry (Becton Dickinson FACS Calibur flow-cytometer) (BD, San Jose, CA, USA). The emission wavelength was detected through the FL1 channel. Data were acquired and analyzed with CellQuest software.

### Preparation of cytosolic and mitochondrial extracts

Cytosolic and mitochondrial extracts were prepared as described previously [72]. MCF-7 and MDA-MB-468 cells were seeded in 90-mm cell culture plates for 1 day, and treated as indicated. Cells were then harvested and washed in PBS. After spinning down, cells were resuspended in 100 μl of HED buffer (10 mM HEPES pH 7.9, 10 mM Kcl, 0.1 mM EDTA pH 8, 1.0 mM dithiothreitol (DTT)) containing 0.4% Nonidet P-40, 1 mM phenylmethylsulfonyl fluoride (PMSF), protease cocktail inhibitor), After incubation on ice for 20–30 min, cell suspensions were vortexed for 10 sec for cell lysis, followed by centrifugation at 5000 rpm for 5 min at 4°C. Cytosolic protein (supernatant) was collected and further centrifuged at 10000 rpm, 30 min to remove crude membranes and to obtain a clear cytosolic fraction free of membrane debris, and stored at −70°C. Mitochondrial extracts (cell pellet) were then washed with mitochondrial extraction buffer (10 mM HEPES pH 7.4, 200 mM mannitol, 70 mM sucrose, 1 mM EGTA or EDTA pH 8, 1 mM DTT, 1 mM PMSF, protease cocktail inhibitor) to remove any traces of cytosolic extract, and finally lysed with 50 μl of mitochondrial extraction buffer on ice for 60 min with intermittent vortexing. Mitochondrial protein was collected after centrifuging at 15,000 rpm for 30 min at 4°C, aliquot and stored at −70°C.

### Western blot analysis of growth regulatory proteins and apoptosis proteins

Cells were treated with ZD6474 and/or UV-B and then the cells were scraped and lysed in Nonidet P-40 lysis buffer (50 mM Tris HCl pH 8.0, 137 mM sodium chloride, 10% glycerol, 1% Nonidet P-40, 50 mM sodium fluoride, 10 mM EDTA) containing 1 mM sodium vanadate, 1 mM phenylmethylsulfonyl fluoride, and protease cocktail inhibitor for obtaining total cell extracts. Equal amount of cell extracts were separated on a 10% sodium dodecyl sulfate-polyacrylamide electrophoretic gel (SDS-PAGE) and transferred to nitrocellulose membranes, which were blocked with 2% BSA and probed with the appropriate antibodies and secondary antibodies. Membranes were then developed using enhanced chemiluminescence or alkaline phosphatase-based colorimetric methods.

### Caspase-3 and caspase-7 activity assays

Caspase-3 and caspase-7 activity was determined by measuring the absorbance at 405 nm after cleavage of synthetic substrate acetyl-Asp-Glu-Val-Asp p-nitroanilide (Ac-DEVD-pNA) as described previously [73] with some modifications [56]. Cells were treated with ZD6474 and/or UV-B radiation for 48 h, and lysed with buffer (50 mM HEPES, pH 7.4, 5 mM CHAPS, 5 mM DTT, 1 mM PMSF, 20 μg/ml leupeptin), followed by centrifugation at 20,000 g for 15 min at 4°C. The lysates (50 μl) were incubated in 200 μM solution of (Ac-DEVD-pNA) in a reaction buffer (20 mM HEPES, pH 7.4, 2 mM EDTA, 0.1% CHAPS, 5 mM DTT) at 37°C. The reaction was monitored for 1–3 h, and the absorbance was recorded at 405 nm. If the signal was low, the reaction can be monitored for 12–24 h. The formation of pNA was calculated as the difference in the absorbance at 405 nm unit time (min) per unit volume (ml) of sample. The relative levels of pNA formation were normalized against the protein concentration (mg/ml) of each extract to obtain specific activity (μ moles pNA/min/mg).

$$\mathrm{Specific}\;\mathrm{activity}\;\left(µ\;\mathrm{moles}\;\mathrm{pNA}/\min/\mathrm{mg}\right)=\frac{\mathrm{\Delta O}.D}{\epsilon \;x\;t\;x\;v\;x\;c}$$

Where,

ϵ = 10.5

v = volume of sample in ml

t = reaction time in minutes

c = concentration of sample in mg/ml

### *In vitro* wounding (scratch) assay

To test the invasive behavior of treated cells, 1 × 10^5^ cells were plated in 6-well tissue culture plates and grown for 24 h to obtain a confluent monolayer and migration was studied by *in vitro* wounding (scratch) assay [74] with slight modifications. The monolayer was scraped (wounded) in a straight line to create a “wound (scratch)” with a p200 pipette tip. The debris were removed and the edge of the wound (scratch) was made smooth by washing the cells once with 1 ml of the growth medium and then replaced with 3 ml of complete media along with ZD6474 and/or UV-B. Cells were observed 48 h post-treatment. Cells invading the wound (scratch) line were observed under an inverted phase-contrast microscope (Leica Microsystems, Wetzlar, Hesse, Germany). The distances between one sides of the scratch with another were measured after the indicated time intervals using the Leica Qwin software. The distance of each wound (scratch) closure was the measure of wound healing. P-values of wound size were calculated using un-paired t-test between the same treatment group, prior and post treatment. Each experiment was performed three times with triplicate samples.

### Scanning electron microscopy (SEM)

Cells were grown in cover slip at a density of 10,000 cells per cover slip. Cells were treated with ZD6474 and/or UV-B radiation for 1 day. After that Cells were fixed with 3.7% Paraformaldehyde (Merck, Mumbai, Mahrashtra, India) for 30 min, followed by serial dehydration in alcohol (50%, 70%, 75%, 90% , 95% and 100% for 5 min at each step) and finally subjected in 100 μl 1,1,1,3,3,3-Hexamethyldisilazane (HMDS) for critical point drying. Samples were then air dried at room temperature and mounted on stub. Next, they were placed in vacuum chamber of SEM gold coating apparatus and gold was coated at 2.5 kV, 20–25 mA for 120 s. The morphogram (surface features) of the MCF-7 and MDA-MB-468 cells were then observed using a JEOL JSM-5800 Scanning Microscope (Zeol, Peabody, MA, USA) using 20 kV acceleration voltages.

### Immunofluorescence studies

MCF-7 and MDA-MB-468 cells were plated on coverslips in DMEM/F-12 complete medium. After 1 day, cells were treated with 1 μM ZD6474 and/or 25 J/m^2^ UV-B for 1 day. Cells were fixed in 3.7% paraformaldehyde, and permeabilized with 0.1% Triton-X-100 and then blocked in 2% BSA, and stained with FITC phalloidin to visualize F-actin (Life Technologies, Grand Island, NY, USA), counterstained with DAPI as per manufacturer’s instructions. Cells were analyzed by confocal laser scanning microscopy (CLSM) (Olympus FluoView FV1000, Version 1.7.1.0) (Olympus, Tokyo, Japan), using the appropriate wavelength. Images were captured and digitized using FLUOVIEW 1000 (Version 1.2.4.0) imaging software.

### VEGF quantification

Breast cancer cells were treated with ZD6474 and/or UV-B and incubated in incomplete medium for 48 h. The conditioned medium (CM) was collected and kept at −70°C for studying secretory proteins (VEGF, MMPs). The concentration of VEGF in the serum-free CM obtained from cultured cells was measured using commercially available sandwich ELISA kits (R&D Systems Inc., Minneapolis, MN, USA) and according to manufacturers’ instructions and the level of VEGF was reported in ng/ml which is normalized to the number of cells.

### Zymography

Activity of matrix metalloprotease-2 (MMP-2) and matrix metalloprotease-9 (MMP-9) was assessed by gelatin Zymography [75]. Briefly, to prepare serum-free conditioned media (CM), cells were allowed to grow to subconfluence in 35-mm tissue culture dishes in DMEM/F-12 containing 10% FBS. After several washes with serum-free medium, the medium was replaced with DMEM/F-12 containing ZD6474 after treatment with UV-B, and the cultures were incubated for an additional 48 h. The conditioned media were collected and applied to SDS-polyacrylamide gels (7.5% w/v) copolymerized with gelatin (0.1% w/v) and washed twice in renaturation buffer (2.5% Triton-X-100) equilibrated in developing buffer (50 mM Tris-cl pH 8.3, 0.2 M Nacl, 5 mM Cacl_2_, 0.02% Brij-35) for initial 30 min at 37°C, followed by incubation in developing buffer at 37°C for 24 h. Enzyme-digested regions were quantified by QuantityOne® (Version 4.2.1) after data acquisition using GS-800™ Calibrated Densitometer (Bio-Rad, Hercules, CA, USA).

## Abbreviations

EGF: Epidermal growth factor; EGFR: Epidermal growth factor receptor; VEGFR: Vascular endothelial growth factor receptor; TKI: Tyrosine kinase inhibitor; RT: Radio-therapy.

## Competing interests

The authors declare that they have no competing interests.

## Authors’ contributions

SS designed, carried out the experiments and also prepared the draft of the manuscript. SR and AK helped in cell culture, preparation of slides for microscopy, and also correcting the manuscript. MM approved the experimental design, assisting the experiments and help in preparing the final draft of the manuscript. All authors read and approved the final manuscript.

## Supplementary Material

Additional file 1: Figure S1 Influence of ZD6474 on UV-irradiated breast cancer cells. Photomicrograph of (A) MCF-7 and (B) MDA-MB-468 irradiated with different doses of UV-B and/or 5 μM ZD6474. Representative data of three independent experiments. Bars, 100 μM. Click here for file

Additional file 2: Figure S2. Expression of Nuclear excision repair (NER) protein in breast cancer cells. Breast cancer cells were collected and whole cell lysates were prepared, protein was separated by SDS-PAGE and western blotting of indicated protein was performed. β-actin was used as loading control. Click here for file
