# Supplementary figures and images for: Targeted therapy against EGFR and VEGFR using ZD6474 enhances the therapeutic potential of UV-B phototherapy in breast cancer cells
Source: Mol Cancer. 2013 Oct 20;12:122. doi: 10.1186/1476-4598-12-122 (PMC4015769; doi:10.1186/1476-4598-12-122)

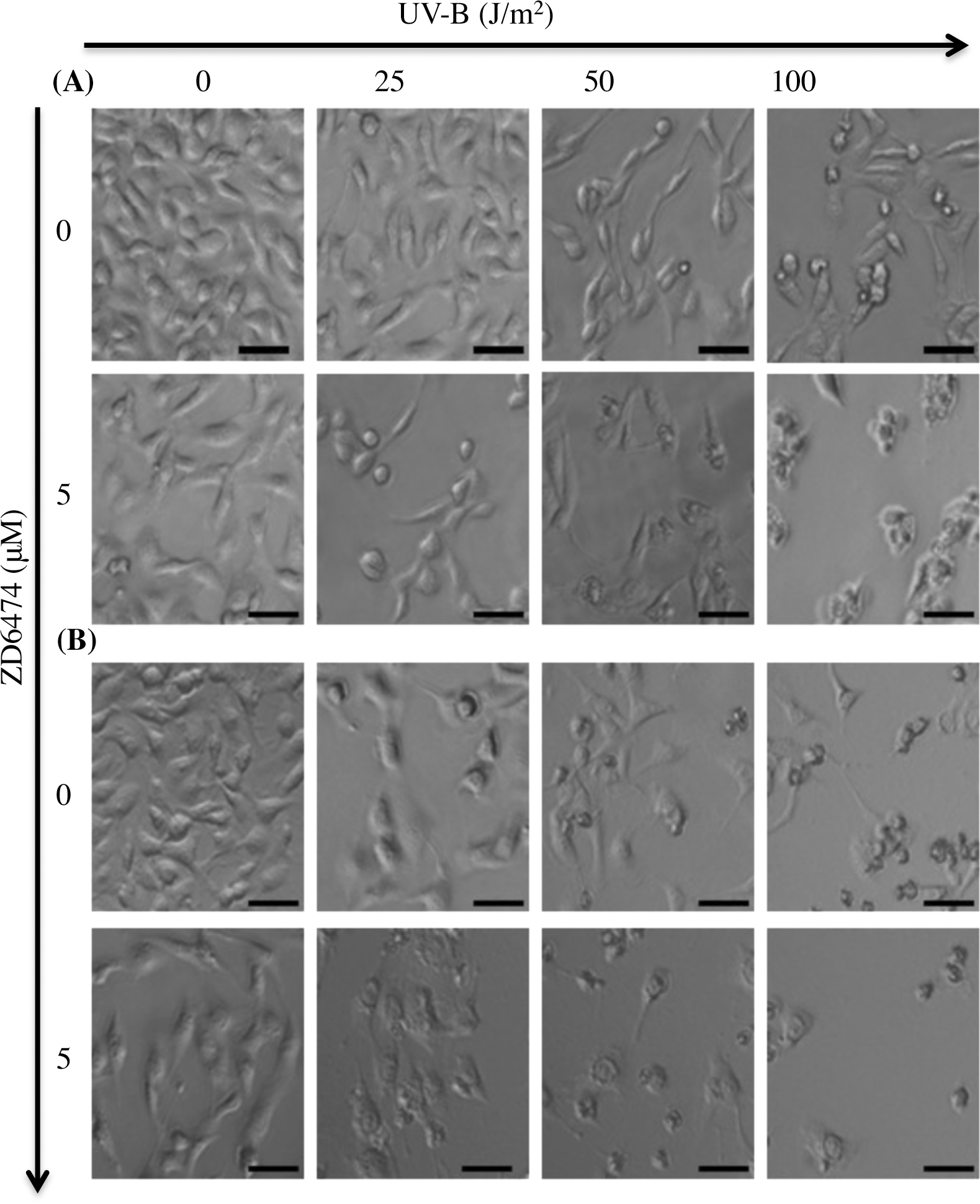

Supplement: Additional file 1: Figure S1 — Influence of ZD6474 on UV-irradiated breast cancer cells. Photomicrograph of (A) MCF-7 and (B) MDA-MB-468 irradiated with different doses of UV-B and/or 5 μM ZD6474. Representative data of three independent experiments. Bars, 100 μM. [file 1476-4598-12-122-S1.tiff]

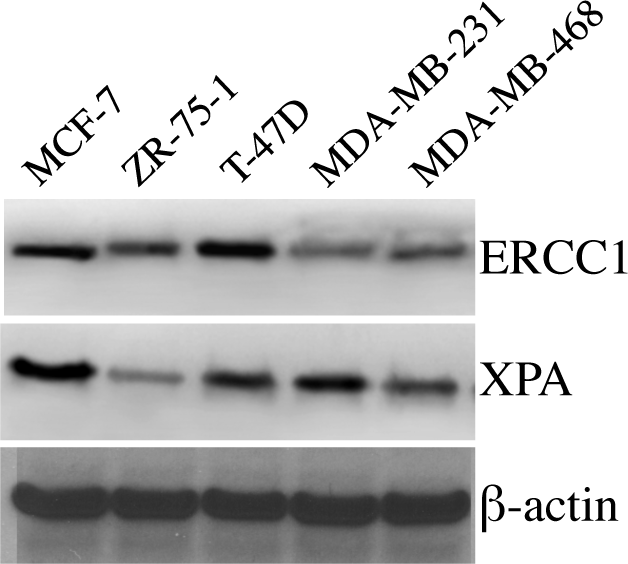

Supplement: Additional file 2: Figure S2. — Expression of Nuclear excision repair (NER) protein in breast cancer cells. Breast cancer cells were collected and whole cell lysates were prepared, protein was separated by SDS-PAGE and western blotting of indicated protein was performed. β-actin was used as loading control. [file 1476-4598-12-122-S2.tiff]
